# Supplementary material for: Development of a humanized anti-FABP4 monoclonal antibody for potential treatment of breast cancer
Source: Breast Cancer Res. 2024 Jul 25;26:119. doi: 10.1186/s13058-024-01873-y (PMC11270797; doi:10.1186/s13058-024-01873-y)
Supplement: Supplementary file 1 — Supplementary Material 1 [file 13058_2024_1873_MOESM1_ESM.pdf]

Absolute Antibody Ltd
Wilton Centre
Redcar
TS10 4RF
United Kingdom
Tel: +44 (0)1642 688810
Email: sales@absoluteantibody.com

12G2
cAb6475-10.0

Product Summary

Custom Number: cAb6475-10.0

Description: Monoclonal antibody manufactured using AbAb's Recombinant Platform

Clone Name: 12G2

Isotype: Human IgG1

Molecular weight: 144297.18 Da

Extinction coefficient: 217840 M^{-1} cm^{-1} (calculation performed as in Pace et al, 1995)

Product Form and Purification

Purification: Purified by affinity chromatography.

Supplied in: PBS

Storage recommendation: Recommended storage at 4°C for up to 1 month. For longer term storage store at -20°C or -80ºC in appropriate sized aliquots.

Lot no: T2106A12

Concentration: 1 mg/ml

Quantity: 21 mg

SDS-PAGE gel image:

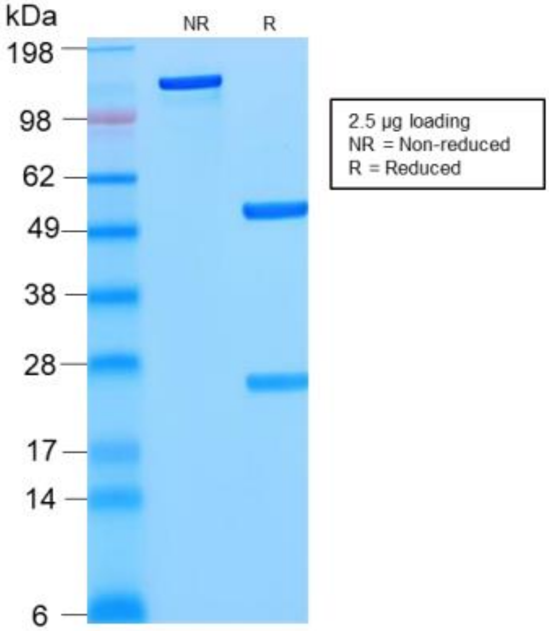

Absolute Antibody Ltd
Wilton Centre
Redcar
TS10 4RF
United Kingdom
Tel: +44 (0)1642 688810
Email: sales@absoluteantbody.com

6H10
cAb6476-10.0

Product Summary

Custom Number: cAb6476-10.0

Description: Monoclonal antibody manufactured using AbAb's Recombinant Platform

Clone Name: 6H10

Isotype: Human IgG1

Molecular weight: 145756.5 Da

Extinction coefficient: 215780 M^{-1} cm^{-1} (calculation performed as in Pace et al, 1995)

Product Form and Purification

Purification: Purified by affinity chromatography.

Supplied in: PBS

Storage recommendation: Recommended storage at 4°C for up to 1 month. For longer term storage store at -20°C or -80°C in appropriate sized aliquots.

Lot no: T2106A13

Concentration: 1 mg/ml

Quantity: 20 mg

SDS-PAGE gel image:

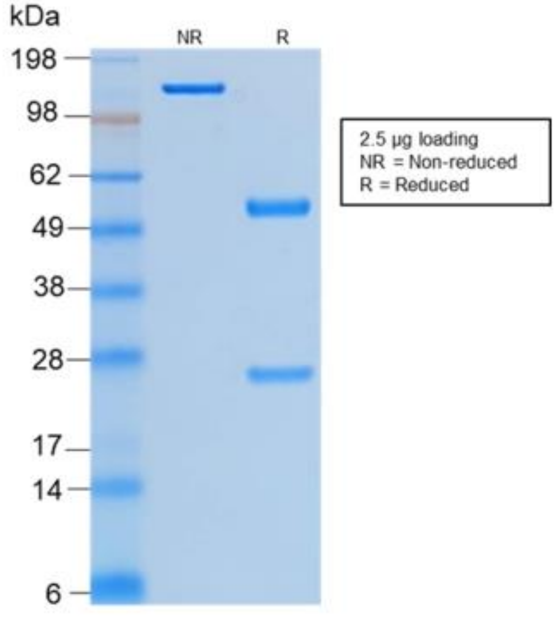

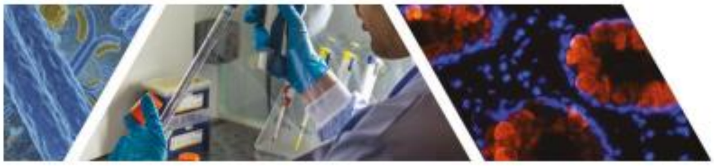

5.2 Antibody expression and purification

Antibodies were expressed and purified by Protein A. Purified protein was buffer exchanged and concentrated. All antibodies expressed and all the purified products looked as expected under non-reducing and reducing SDS-PAGE (Figure 3).

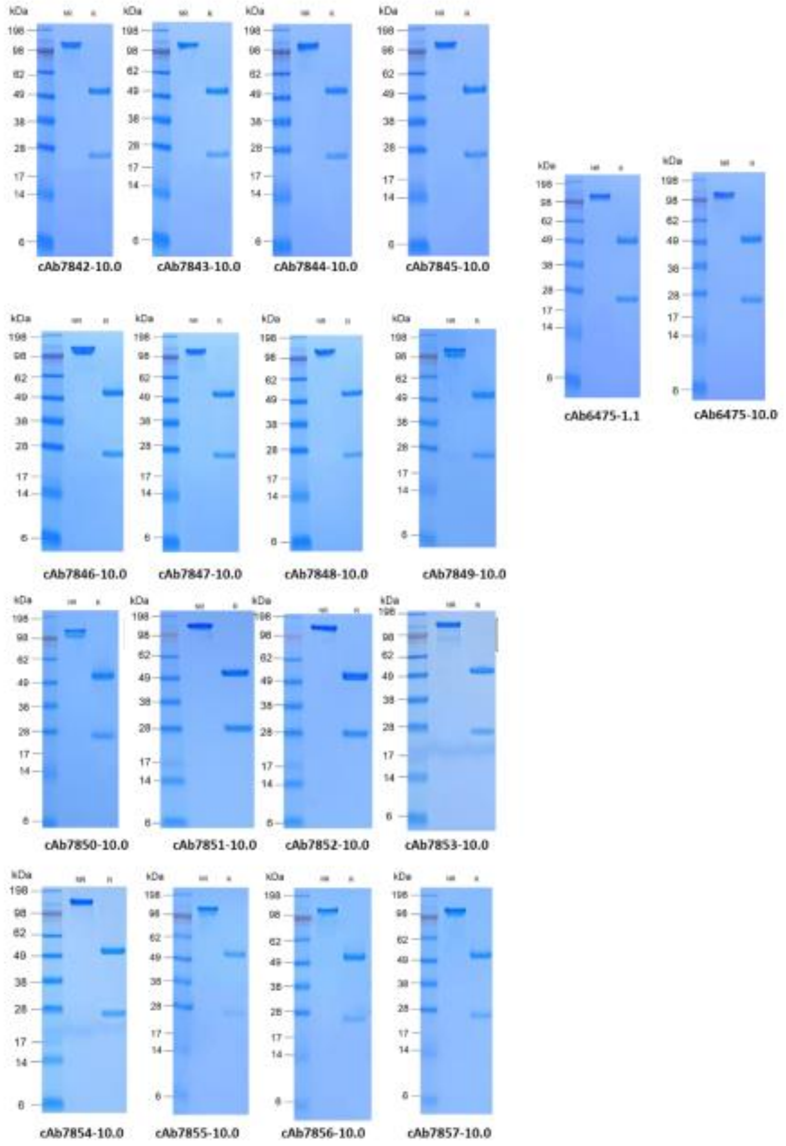

Figure 3. SDS-PAGE gels of purified antibodies. All antibodies are shown in non-reducing (NR) and reducing (R) conditions. 2.5 μg of protein was loaded in each lane.
