# Supplementary material for: Development of a humanized anti-FABP4 monoclonal antibody for potential treatment of breast cancer
Source: Breast Cancer Res. 2024 Jul 25;26:119. doi: 10.1186/s13058-024-01873-y (PMC11270797; doi:10.1186/s13058-024-01873-y)
Supplement: Supplementary file 2 — Supplementary Material 2 [file 13058_2024_1873_MOESM2_ESM.docx]

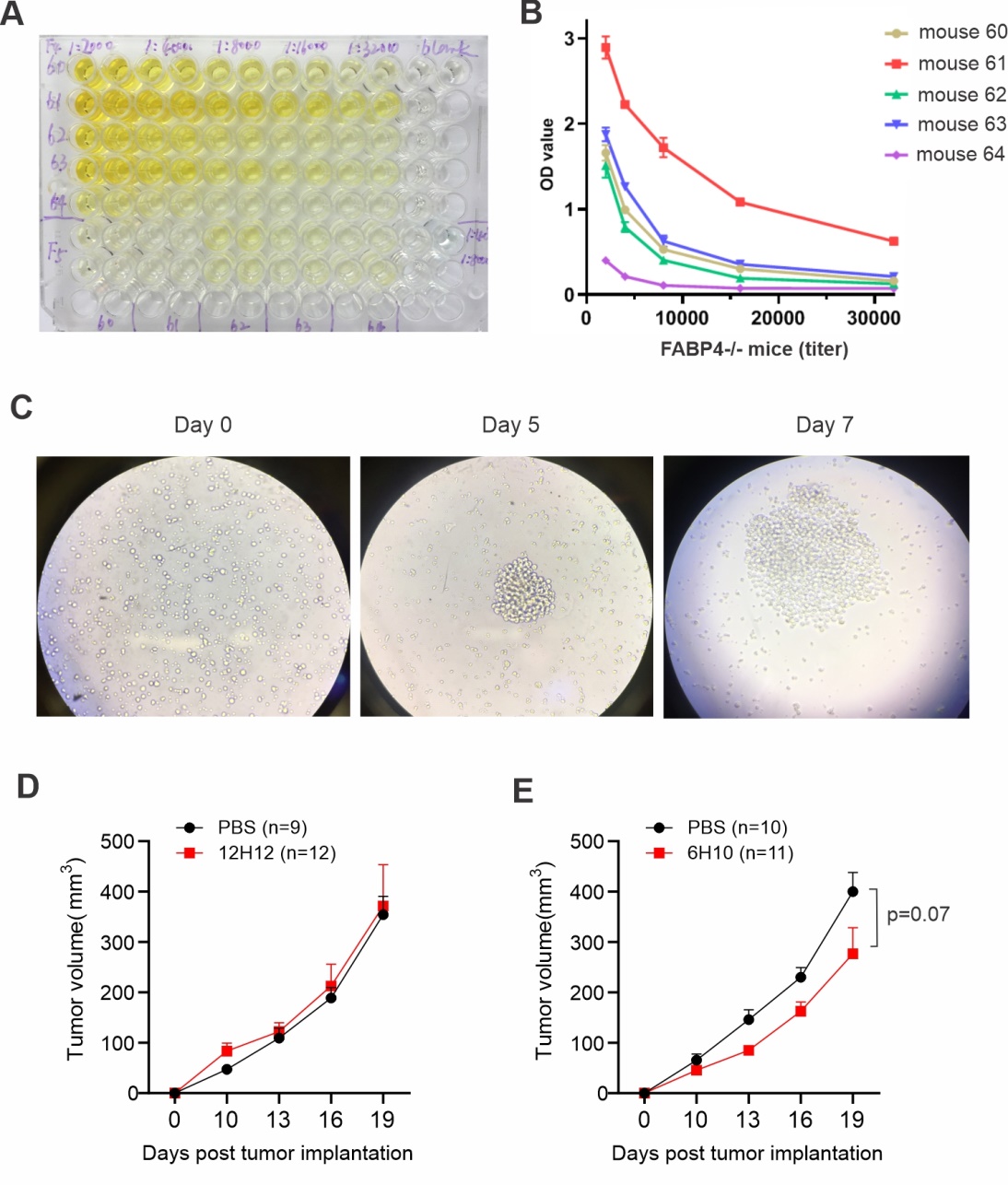


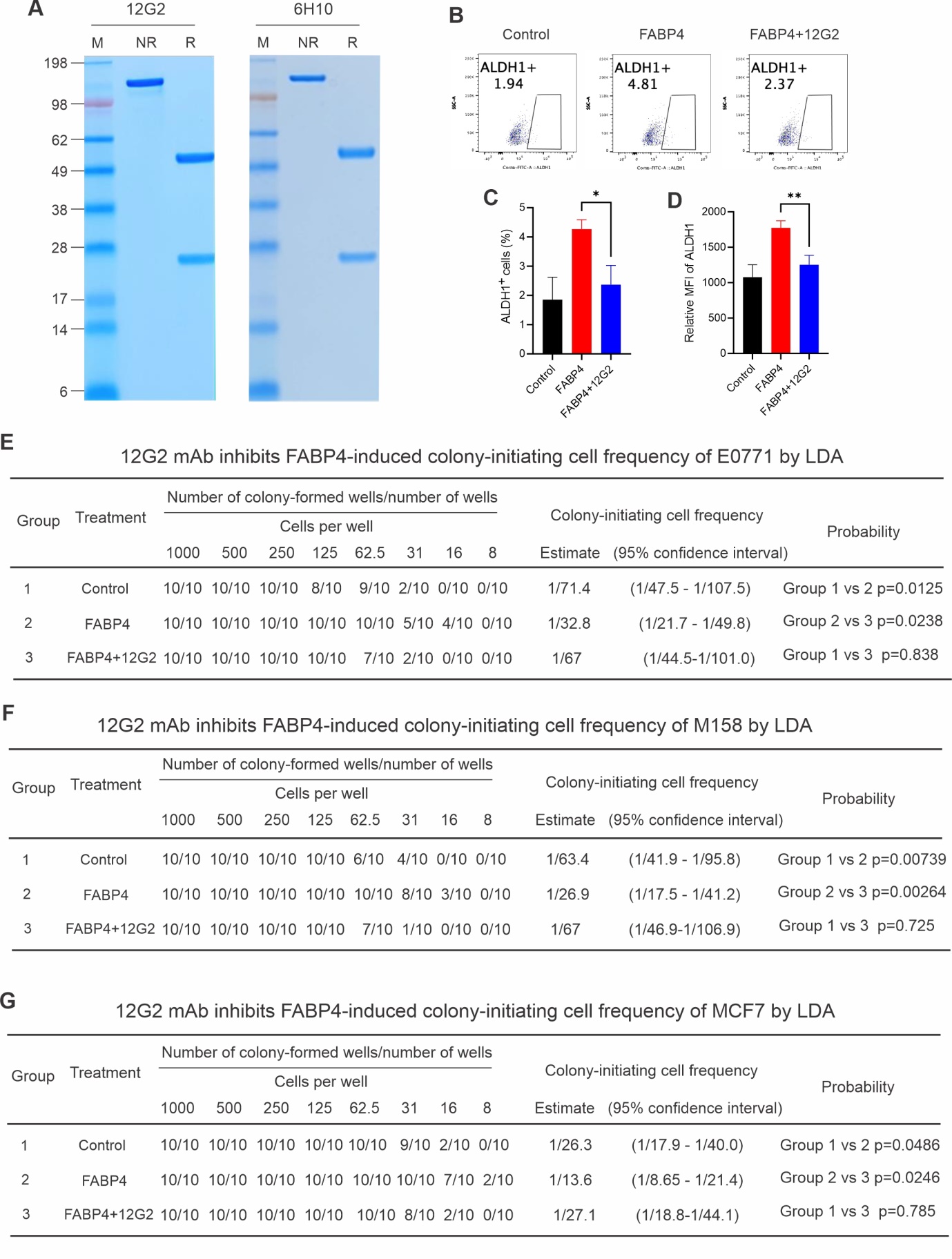

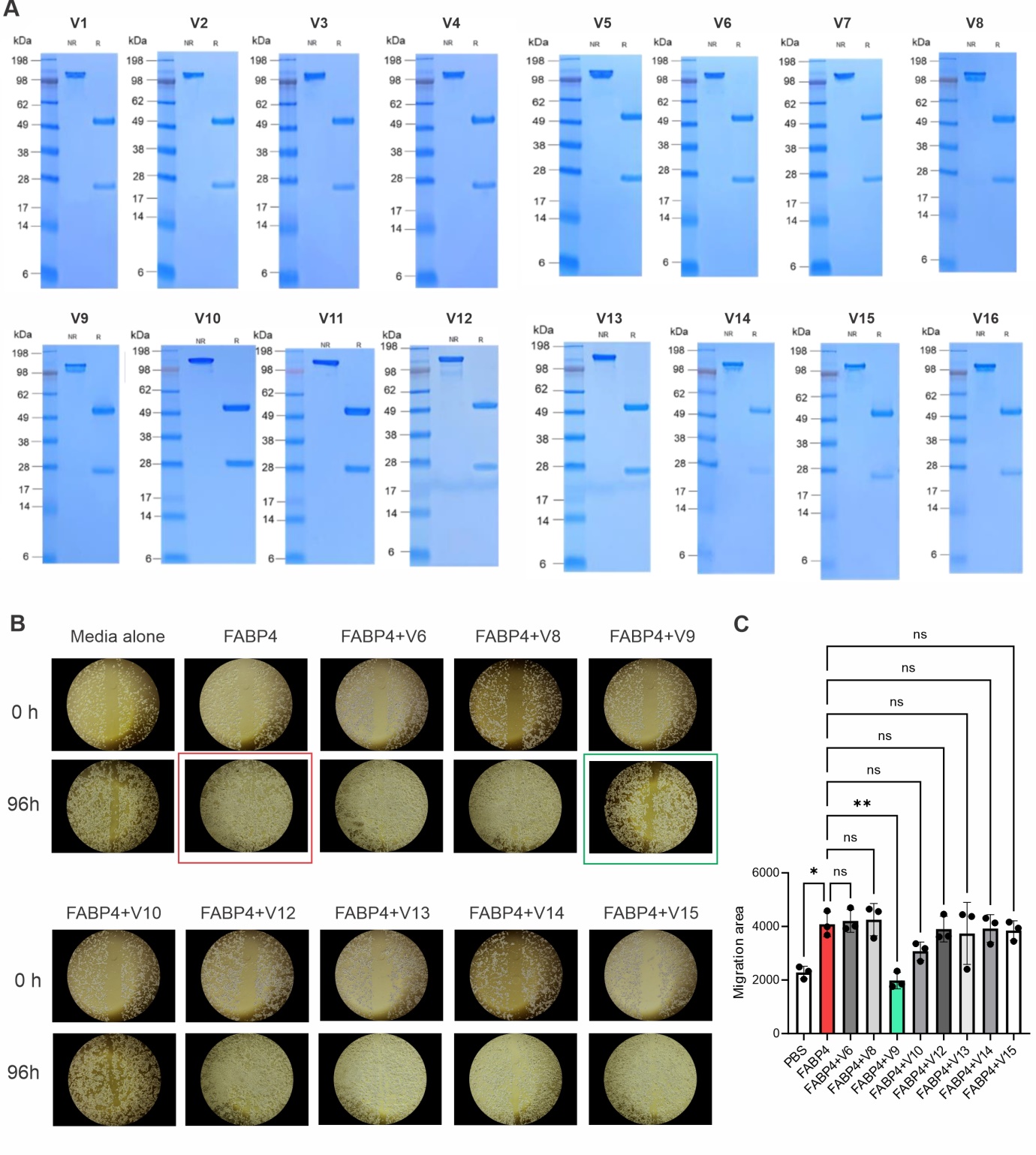

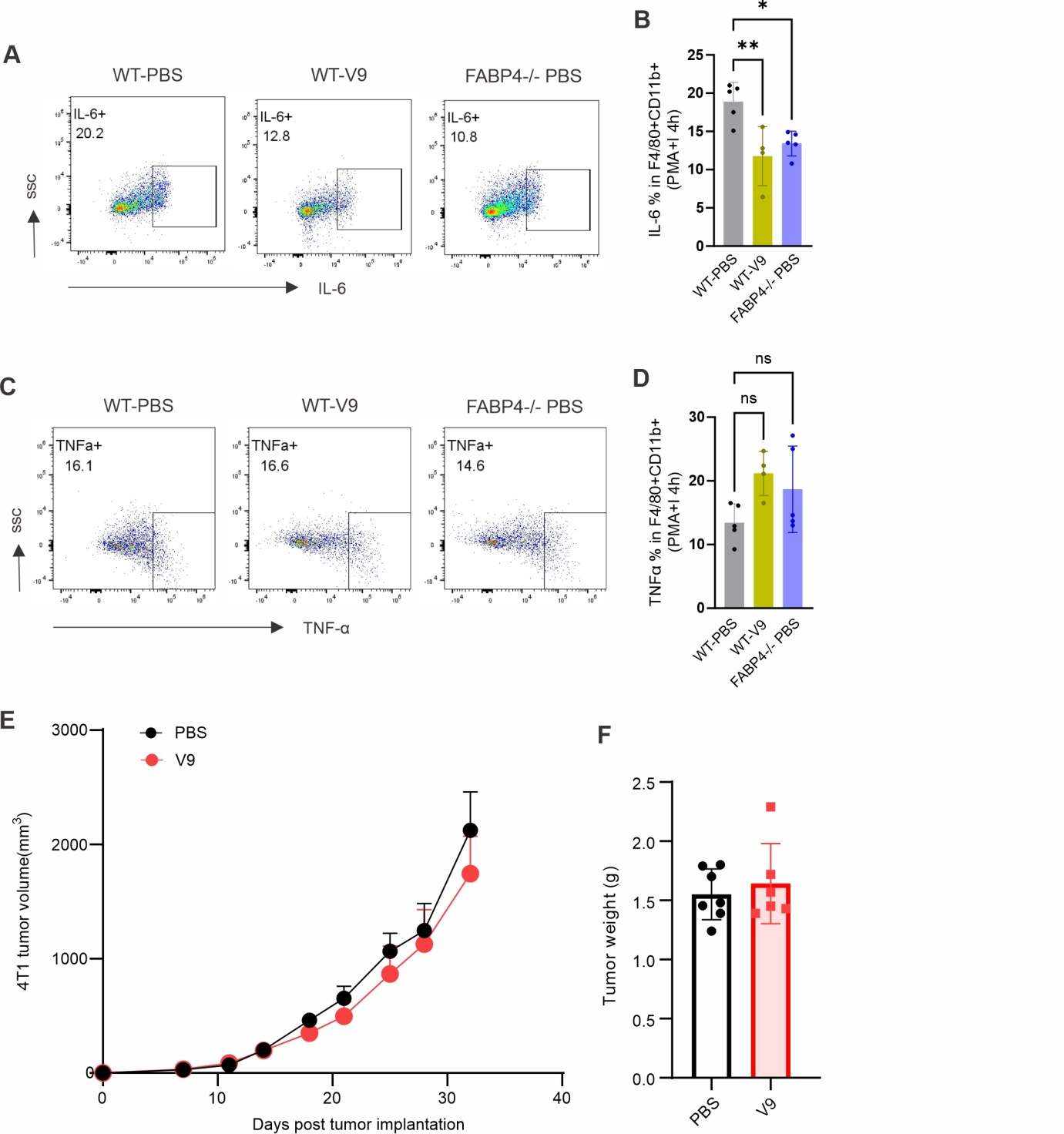

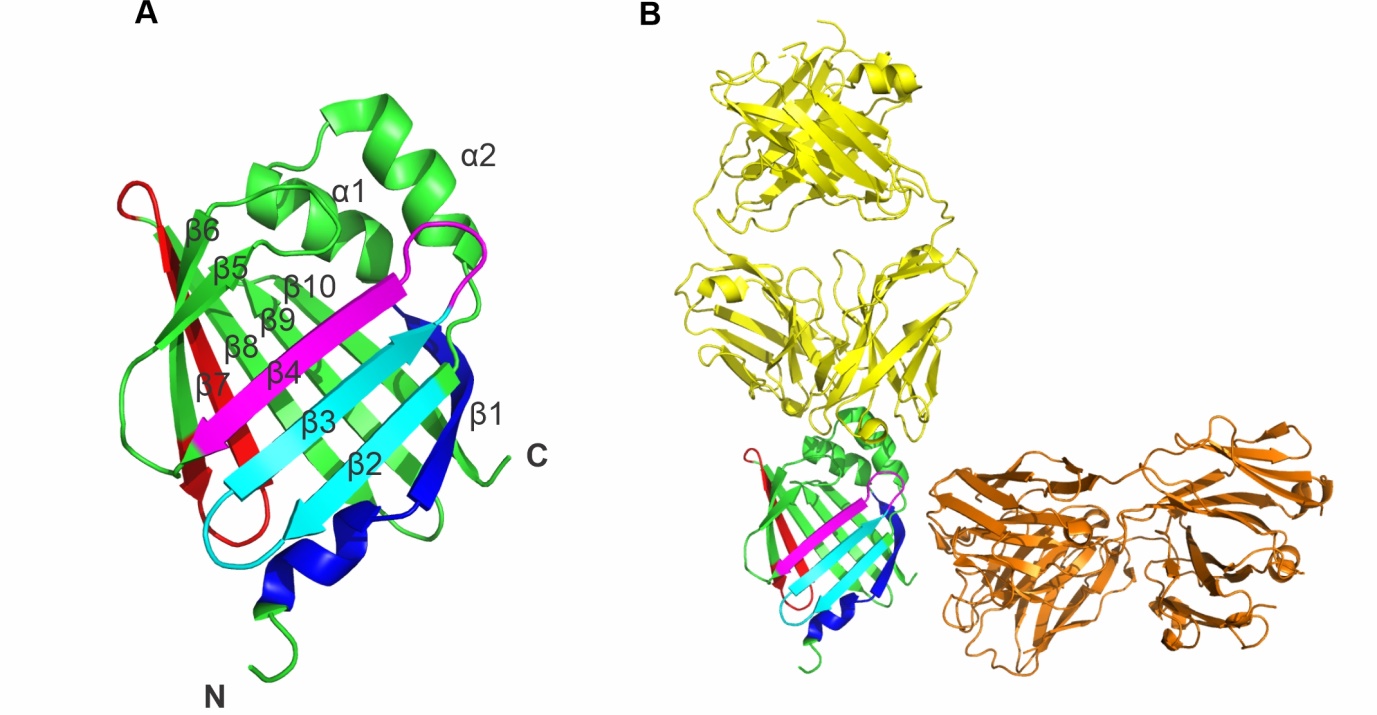

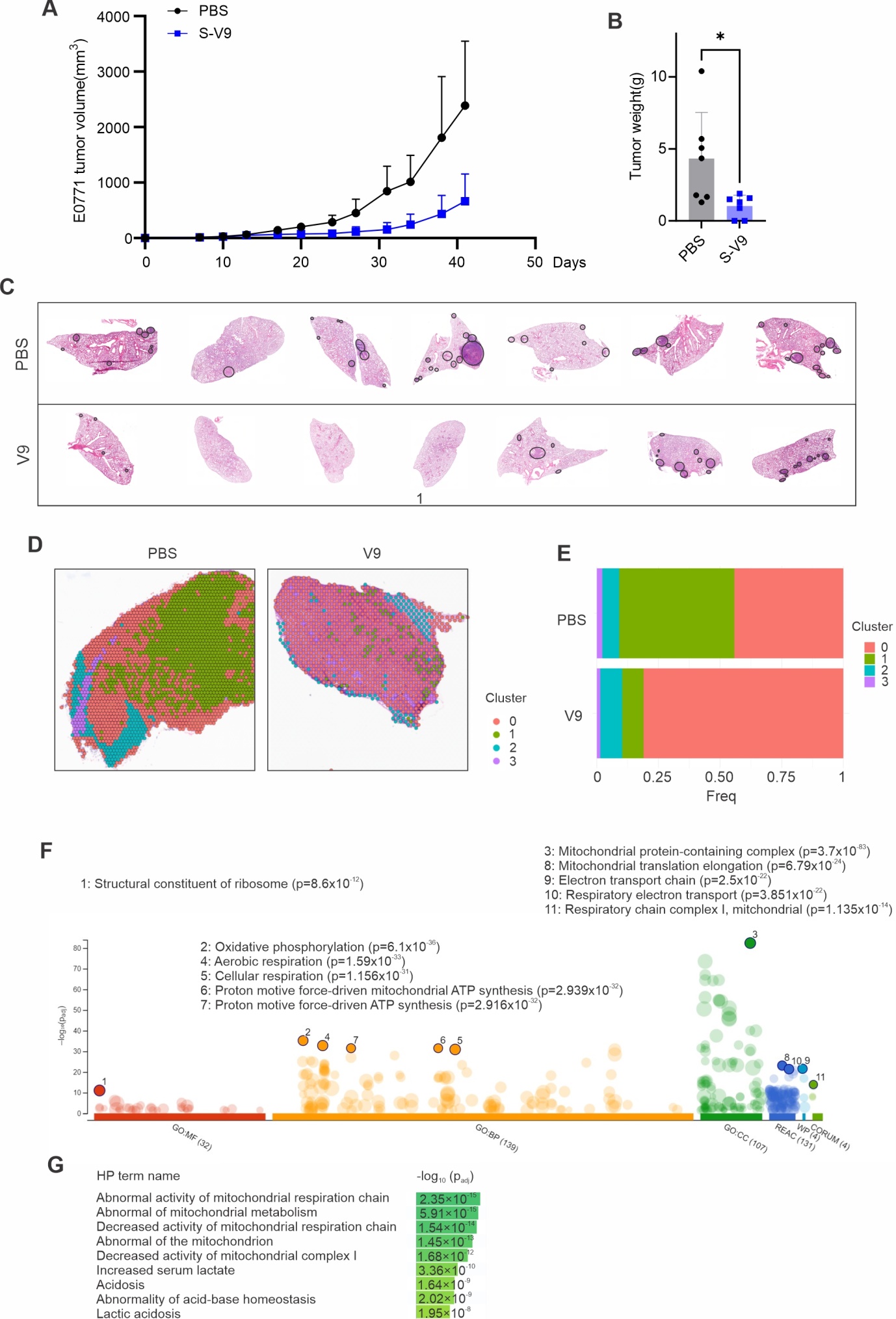
**Supplementary Figure 1 Screening of anti-FABP4 hybridoma clones for the treatment of mammary tumors**

(A,B) Evaluating anti-FABP4 serum titer in FABP4^-/-^ mice immunized with recombinant human FABP4 protein. The OD value of anti-FABP4 titer in diluted serum is shown in panel B.

(C) Monoclonal hybridoma cells were developed and selected for testing of specific binding to FABP4.

(D,E) mAbs from 12H2 (D) and 6H10 (E) clones were purified and used for the treatment of E0771 tumors in mouse models. Antibody treatment (30mg/kg) started from day 7 post tumor injection.

**Supplementary Figure 2 Assessing blocking activity of anti-FABP4 antibodies in vitro**

1. Analysis of purity of the 12G2 and 6H10 antibodies by SDS-PAGE under non-reducing (NR) and reducing (R) conditions.

(B-D) MCF-7 cells were treated with PBS control, recombinant human FABP4 (100ng/ml) or recombinant human FABP4+12G2 for 24 hours. ALDH1 activity were measured by flow cytometric staining. Percentage (C) and mean fluorescent intensity (D) of ALDH1^+^ cells are shown in panel C and D, respectively (*p<0.05, **p<0.01).

(E-G) Limiting dilution assays were used to determine the blocking activity of 12G2 in inhibiting FABP4-induced colony-initiating cell frequency using E0771 cells (E), M158 cells (F) and MCF7 cells (G).

**Supplementary Figure 3 Evaluating the blocking activity of humanized anti-FABP4 antibodies in vitro**

1. Analysis of the purity of 16 humanized 12G2 variants (V1-V16) by SDS-PAGE under non-reducing (NR) and reducing (R) conditions.

(B, C) MCF-7 cells were cultured in medium alone, recombinant human FABP4 alone (100ng/ml) (red square), or recombinant human FABP4+individual humanized antibody variants, for 96 hours. Wound healing assay was conducted to determine that V9 antibody variant (green square) was able to inhibit FABP4-induced MCF-7 migration. Migration area in each group is shown in panel C (* p<0.05, **p<0.01, ns, non-significant)

**Supplementary Figure 4 Analysis of the impact of V9 mAb treatment on tumor infiltrating associated macrophages.**

(A,B) Intracellular staining of IL-6 production in tumor infiltrating macrophages in tumors from mice treated with either PBS or V9 antibody. The percentage of IL-6^+^ macrophages is shown in panel B (*p<0.05, **p<0.01).

(C,D) Intracellular staining of TNFα production in tumor infiltrating macrophages in tumors from mice treated with either PBS or V9 antibody. The percentage of TNF-α^+^ macrophages is shown in panel B (ns, non-significant).

(E,F) 4T1 tumor growth curve in Balb/c mice treated with PBS or V9 mAb (10mg/kg). Tumor weight on day 33 post tumor implantation is shown in panel F.

**Supplementary Figure 5** **Analysis of V9 mAb binding epitopes within the three dimensional structural of FABP4**

(A) Secondary structure image of V9 binding to β-1 (blue), β-2/3 (cyan), β-3/4 (magenta), and β-7 (red) in the structure of FABP4 (PDB 6LJW).

(B) Computer modeling of unique binding sites of V9 (magenta) to FABP4 (PDB 5D8J/5C0N) in comparison with other FABP4 antibodies, including CA33 (green) and HA3 (cyan).

**Supplementary Figure 6 S-V9 mAb treatment inhibited E0771 tumor growth and metastasis**

(A, B) E0771 tumor growth curve in mice treated with PBS or S-V9 mAb (5mg/kg) for 6 weeks. Tumor weight is shown in panel B (*p<0.05).

(C) Analysis of metastatic nodules (black circle) in lungs of mice treated with PBS or S-V9 mAb by H&E staining.

(D) Spatial Dimplot showing unsupervised cell clusters in tumors treated with PBS or S-V9 antibody, respectively.

(E) Cluster proportions in tumors treated with PBS or S-V9 antibody, respectively.

(F) g:Profiler pathway analysis, including gene ontology (GO), KEGG Reactome (REAC), WikePathways (WP), protein complexes from CORUM using cluster 1 DEGs.

(G) g:Profiler analysis of human disease phenotypic (HP) using cluster 1 DEGs.

**Table S1 Selected mAb specific binding to FABP4**

| **Clones** | **Affinity to FABP4** | **Affinity to FABP5** | **Ascites production** |
| --- | --- | --- | --- |
| 3B10-1 | +++ | - | Yes |
| 3B10-2 | +++ | - | no |
| 3B10-3 | +++ | - | few |
| 5A9-1 | +++ | - | no |
| 5A9-2 | +++ | - | few |
| 5A9-3 | +++ | - | no |
| 6D5 | ++ | - | no |
| 6F12 | + | - | few |
| 9G4-1 | +++ | - | Yes |
| 7A12 | + | - | few |
| 12G2 | ++ | - | Yes |
| 12G7 | ++ | - | few |
| 12A3 | + | - | no |
| 12B1-2 | + | - | few |
| 12B4-1 | ++ | - | few |
| 12H12 | ++ | - | Yes |
| 9D4 | ++ | - | Yes |
| 1A2 | ++ | - | few |
| 1F6 | + | - | few |
| 3C4 | + | - | few |
| 3H10 | +++ | - | few |
| 4G4 | +++ | - | few |
| 6H10 | +++ | - | Yes |
| 13F2 | +++ | - | few |
| 13G7 | ++ | - | few |

Binding affinity: +++ best, ++ better, + good, - negative

**Table S2. Synthesized biotinylated FABP4 peptides**

| Peptide No | N-Term | Sequence | C-Term | Hydro | MWt |
| --- | --- | --- | --- | --- | --- |
| 1 | H- | MCDAFVGTWKLVSSE(261) | -NH2 | 0.413 | 2026.43 |
| 2 | Biotin- | SGSGVGTWKLVSSENFDDY | -NH2 | 0.327 | 2273.47 |
| 3 | Biotin- | SGSGLVSSENFDDYMKEVG | -NH2 | 0.235 | 2246.47 |
| 4 | Biotin- | SGSGNFDDYMKEVGVGFAT | -NH2 | 0.301 | 2206.45 |
| 5 | Biotin- | SGSGMKEVGVGFATRKVAG | -NH2 | 0.25 | 2063.42 |
| 6 | Biotin- | SGSGVGFATRKVAGMAKPN | -NH2 | 0.242 | 2060.42 |
| 7 | Biotin- | SGSGRKVAGMAKPNMIISV | -NH2 | 0.358 | 2128.62 |
| 8 | Biotin- | SGSGMAKPNMIISVNGDVI | -NH2 | 0.459 | 2115.53 |
| 9 | Biotin- | SGSGMIISVNGDVITIKSE | -NH2 | 0.446 | 2132.49 |
| 10 | Biotin- | SGSGNGDVITIKSESTFKN | -NH2 | 0.18 | 2166.39 |
| 11 | Biotin- | SGSGTIKSESTFKNTEISF | -NH2 | 0.252 | 2245.5 |
| 12 | Biotin- | SGSGSTFKNTEISFILGQE | -NH2 | 0.359 | 2227.48 |
| 13 | Biotin- | SGSGTEISFILGQEFDEVT | -NH2 | 0.428 | 2241.47 |
| 14 | Biotin- | SGSGILGQEFDEVTADDRK | -NH2 | 0.123 | 2249.45 |
| 15 | Biotin- | SGSGFDEVTADDRKVKSTI | -NH2 | 0.104 | 2237.48 |
| 16 | Biotin- | SGSGADDRKVKSTITLDGG | -NH2 | 0.072 | 2089.31 |
| 17 | Biotin- | SGSGVKSTITLDGGVLVHV | -NH2 | 0.488 | 2051.39 |
| 18 | Biotin- | SGSGTLDGGVLVHVQKWDG | -NH2 | 0.393 | 2137.4 |
| 19 | Biotin- | SGSGVLVHVQKWDGKSTTI | -NH2 | 0.398 | 2224.57 |
| 20 | Biotin- | SGSGQKWDGKSTTIKRKRE | -NH2 | -0.083 | 2374.71 |
| 21 | Biotin- | SGSGKSTTIKRKREDDKLV | -NH2 | -0.078 | 2330.68 |
| 22 | Biotin- | SGSGKRKREDDKLVVECVM | -NH2 | 0.078 | 2361.8 |
| 23 | Biotin- | SGSGDDKLVVECVMKGVTS | -NH2 | 0.32 | 2136.5 |
| 24 | Biotin- | SGSGVECVMKGVTSTRVYE | -NH2 | 0.339 | 2214.57 |
| 25 | Biotin- | SGSGCVMKGVTSTRVYERA | -OH | 0.27 | 2214.58 |

**Table S3: Marker genes of cluster 0 and 1**

| Cluster | p_val | avg_log2FC | pct.1 | pct.2 | p_val_adj | gene |
| --- | --- | --- | --- | --- | --- | --- |
| 0 | 0.00E+00 | 0.9368257 | 0.998 | 0.801 | 0 | Rps13 |
| 0 | 0.00E+00 | 0.9494845 | 0.999 | 0.809 | 0 | Rpl27a |
| 0 | 0.00E+00 | 0.984324 | 1 | 0.91 | 0 | Rps12 |
| 0 | 0.00E+00 | 0.8994034 | 1 | 0.966 | 0 | Fau |
| 0 | 0.00E+00 | 0.9257794 | 1 | 0.983 | 0 | Rpl13 |
| 0 | 0.00E+00 | 0.9487995 | 0.995 | 0.712 | 0 | Mif |
| 0 | 0.00E+00 | 0.8937152 | 1 | 0.826 | 0 | Rpl36 |
| 0 | 0.00E+00 | 1.0102008 | 0.855 | 0.302 | 0 | 1110038B12Rik |
| 0 | 0.00E+00 | 1.099856 | 0.889 | 0.345 | 0 | Gm11808 |
| 0 | 0.00E+00 | 1.192708 | 0.831 | 0.296 | 0 | Fabp5 |
| 0 | 0.00E+00 | 0.94053 | 0.92 | 0.391 | 0 | Tma7 |
| 0 | 0.00E+00 | 0.9457118 | 0.597 | 0.144 | 0 | Lmo1 |
| 0 | 0.00E+00 | 0.9014207 | 0.596 | 0.149 | 0 | Cks2 |
| 0 | 0.00E+00 | 1.1738808 | 0.863 | 0.342 | 0 | Gm10076 |
| 0 | 0.00E+00 | 1.0073564 | 0.557 | 0.129 | 0 | Gm43154 |
| 0 | 0.00E+00 | 0.8968978 | 0.63 | 0.185 | 0 | Snhg8 |
| 0 | 0.00E+00 | 1.3661251 | 0.45 | 0.087 | 0 | Ccl4 |
| 0 | 0.00E+00 | 1.6178051 | 0.415 | 0.075 | 0 | Mmp12 |
| 0 | 0.00E+00 | 0.9512724 | 0.485 | 0.125 | 0 | Ube2a |
| 0 | 0.00E+00 | 1.06225 | 0.369 | 0.06 | 0 | Spink2 |
| 0 | 0.00E+00 | 0.9106443 | 0.426 | 0.093 | 0 | Il1b |
| 0 | 0.00E+00 | 0.9022906 | 0.462 | 0.122 | 0 | Hilpda |
| 0 | 0.00E+00 | 0.9589917 | 0.468 | 0.127 | 0 | Adm |
| 0 | 0.00E+00 | 0.97771 | 0.377 | 0.072 | 0 | Pdcd2 |
| 0 | 0.00E+00 | 0.9866003 | 0.342 | 0.055 | 0 | Ttc32 |
| 0 | 0.00E+00 | 0.944532 | 0.414 | 0.1 | 0 | Lsm12 |
| 0 | 0.00E+00 | 1.0298167 | 0.438 | 0.117 | 0 | Bcl2a1b |
| 0 | 0.00E+00 | 0.9484294 | 0.315 | 0.048 | 0 | Rhox1 |
| 0 | 0.00E+00 | 0.9566189 | 0.393 | 0.096 | 0 | Churc1 |
| 0 | 0.00E+00 | 1.1162666 | 0.334 | 0.101 | 0 | S100a8 |
| 1 | 0.00E+00 | 3.23305 | 0.897 | 0.85 | 0 | Ivns1abp |
| 1 | 0.00E+00 | 2.8148569 | 0.95 | 0.942 | 0 | Ewsr1 |
| 1 | 0.00E+00 | 2.6653159 | 0.996 | 0.999 | 0 | Rpl5 |
| 1 | 0.00E+00 | 2.890204 | 0.998 | 0.999 | 0 | Rpl4 |
| 1 | 0.00E+00 | 2.613298 | 0.767 | 0.84 | 0 | Vmp1 |
| 1 | 0.00E+00 | 2.652628 | 0.698 | 0.774 | 0 | Vps4b |
| 1 | 0.00E+00 | 2.5648621 | 0.672 | 0.72 | 0 | 2410004B18Rik |
| 1 | 0.00E+00 | 2.5937116 | 0.638 | 0.72 | 0 | Crk |
| 1 | 0.00E+00 | 2.6665169 | 0.618 | 0.694 | 0 | Pck2 |
| 1 | 0.00E+00 | 3.0906692 | 0.546 | 0.563 | 0 | Parp14 |
| 1 | 0.00E+00 | 2.8084533 | 0.544 | 0.59 | 0 | Mark3 |
| 1 | 0.00E+00 | 3.4168765 | 0.449 | 0.374 | 0 | Csprs |
| 1 | 0.00E+00 | 2.79001 | 0.531 | 0.594 | 0 | Leo1 |
| 1 | 0.00E+00 | 2.5821561 | 0.492 | 0.606 | 0 | Txndc15 |
| 1 | 0.00E+00 | 2.5530415 | 0.443 | 0.558 | 0 | Tm7sf3 |
| 1 | 0.00E+00 | 2.5693818 | 0.439 | 0.552 | 0 | Eif2b3 |
| 1 | 0.00E+00 | 2.6409359 | 0.425 | 0.528 | 2E-07 | Smc3 |
| 1 | 0.00E+00 | 2.8426031 | 0.382 | 0.437 | 4E-07 | Sh2d5 |
| 1 | 0.00E+00 | 2.5883418 | 0.428 | 0.551 | 2.21E-05 | Ryk |
| 1 | 0.00E+00 | 2.5907065 | 0.419 | 0.54 | 0.00017 | Nob1 |
| 1 | 0.00E+00 | 2.6181509 | 0.411 | 0.518 | 0.000173 | Pqlc3 |
| 1 | 0.00E+00 | 2.6546552 | 0.379 | 0.456 | 0.000479 | Mfn1 |
| 1 | 1.00E-07 | 2.793744 | 0.355 | 0.416 | 0.003516 | Ckap2 |
| 1 | 8.00E-07 | 2.5480239 | 0.387 | 0.486 | 0.024677 | Srek1 |
| 1 | 1.50E-06 | 2.7564053 | 0.35 | 0.416 | 0.049061 | Srpk2 |
| 1 | 1.60E-06 | 2.5842248 | 0.397 | 0.515 | 0.052952 | Csnk1g3 |
| 1 | 4.60E-06 | 2.8382481 | 0.312 | 0.35 | 0.149621 | Gtpbp2 |
| 1 | 1.07E-05 | 2.7692026 | 0.351 | 0.428 | 0.344586 | Acadm |
| 1 | 3.13E-05 | 2.6569848 | 0.366 | 0.462 | 1 | Dnajc21 |
| 1 | 5.53E-05 | 2.6253768 | 0.365 | 0.462 | 1 | Insl6 |

**Table S4: Diseases associated to differentially expressed genes (DEGs) in cluster 1 by iPathwayGuide analysis**

| **Disease Name** | **countDE** | **countAll** | **pv** |
| --- | --- | --- | --- |
| Mitochondrial complex I deficiency | 10 | 42 | 7.77E-13 |
| Combined oxidative phosphorylation deficiency | 8 | 61 | 2.38E-08 |
| Hypomyelinating leukodystrophy; Pelizaeus-Merzbacher disease (PMD) | 5 | 34 | 6.10E-06 |
| Dilated cardiomyopathy | 5 | 52 | 5.09E-05 |
| Viral myocarditis | 3 | 10 | 5.22E-05 |
| Dermatitis herpetiformis | 3 | 10 | 5.22E-05 |
| Vogt-Koyanagi-Harada syndrome; Vogt-Koyanagi-Harada disease; Uveomeningoencephalitic syndrome | 3 | 10 | 5.22E-05 |
| Hashimoto thyroiditis | 3 | 11 | 7.13E-05 |
| Graves disease | 3 | 11 | 7.13E-05 |
| Eosinophilic granulomatosis with polyangiitis; Churg-Strauss syndrome | 3 | 11 | 7.13E-05 |
| Multiple sclerosis | 3 | 12 | 9.45E-05 |
| Sjogren syndrome | 3 | 12 | 9.45E-05 |
| Adult onset Still disease; Adult Still disease | 3 | 12 | 9.45E-05 |
| Giant cell arteritis; Temporal arteritis | 3 | 13 | 0.000122 |
| Celiac disease | 3 | 13 | 0.000122 |
| Mohr-Tranebjaerg syndrome | 2 | 3 | 0.000176 |
| Jensen syndrome; Opticoacoustic nerve atrophy | 2 | 3 | 0.000176 |
| Primary central nervous system lymphoma | 3 | 15 | 0.000192 |
| Tuberculosis | 3 | 16 | 0.000235 |
| Asthma | 3 | 18 | 0.000339 |
| Systemic lupus erythematosus | 3 | 20 | 0.000468 |
| Allograft rejection | 3 | 21 | 0.000543 |
| Systemic sclerosis; Systemic scleroderma | 3 | 23 | 0.000714 |
| Oocyte maturation defect | 3 | 24 | 0.000812 |
| Mitochondrial complex III deficiency | 2 | 9 | 0.002052 |
| Type 1 diabetes mellitus | 3 | 35 | 0.002466 |
| Dyskeratosis congenita | 2 | 11 | 0.003102 |
| Congenital symmetric circumferential skin creases; Kunze-Riehm syndrome; Michelin tire baby syndrome | 2 | 11 | 0.003102 |
| Congenital fibrosis of the extraocular muscles | 2 | 13 | 0.004355 |
| Cleft lip and/or cleft palate | 2 | 15 | 0.005804 |
| Keratosis linearis with ichthyosis congenita and sclerosing keratoderma; KLICK syndrome | 1 | 1 | 0.007694 |
| P14 deficiency | 1 | 1 | 0.007694 |
| Ethylmalonic encephalopathy | 1 | 1 | 0.007694 |
| Autoinflammation lipodystrophy and dermatosis syndrome; Proteasome associated autoinflammatory syndromes (PRAAS); Chronic atypical neutrophilic dermatosis with lipodystrophy and elevated temperature (CANDLE) syndrome; Joint contractures, muscle atrophy, microcytic anemia, and panniculitis-induced lipodystrophy (JMP); Japanese autoinflammatory syndrome with lipodystrophy (JASL) | 1 | 1 | 0.007694 |
| Spondylometaphyseal dysplasia, Sedaghatian type | 1 | 1 | 0.007694 |
| Burn-McKeown syndrome | 1 | 1 | 0.007694 |
| Cerebrocostomandibular syndrome | 1 | 1 | 0.007694 |
| Retinal dystrophy, iris coloboma, and comedogenic acne syndrome | 1 | 1 | 0.007694 |
| Leigh syndrome | 2 | 18 | 0.008329 |
| Noonan syndrome and related disorders | 2 | 19 | 0.009262 |
| Myelodysplastic syndrome | 2 | 19 | 0.009262 |
| Juvenile-onset dystonia | 1 | 2 | 0.015328 |
| Nestor-Guillermo progeria syndrome | 1 | 2 | 0.015328 |
| Baraitser-Winter syndrome | 1 | 2 | 0.015328 |
| MEDNIK syndrome; Erythrokeratodermia variabilis type 3 | 1 | 2 | 0.015328 |
| Amyloidosis, Finnish type; Meretoja syndrome; Amyloid cranial neuropathy with lattice corneal dystrophy | 1 | 2 | 0.015328 |
| Phosphoenolpyruvate carboxykinase deficiency | 1 | 2 | 0.015328 |
| Myelodysplastic/myeloproliferative neoplasms | 2 | 25 | 0.015763 |
| Osteogenesis imperfecta | 2 | 30 | 0.022288 |
| Complex cortical dysplasia with other brain malformations | 2 | 30 | 0.022288 |

**Table S5: Diseases associated to differentially expressed genes (DEGs) in cluster 0 by iPathwayGuide analysis**

| **Disease Name** | **countDE** | **countAll** | **pv** |
| --- | --- | --- | --- |
| Multiple sclerosis | 6 | 12 | 1.22E-09 |
| Primary central nervous system lymphoma | 6 | 15 | 6.44E-09 |
| Viral myocarditis | 5 | 10 | 3.20E-08 |
| Dermatitis herpetiformis | 5 | 10 | 3.20E-08 |
| Vogt-Koyanagi-Harada syndrome; Vogt-Koyanagi-Harada disease; Uveomeningoencephalitic syndrome | 5 | 10 | 3.20E-08 |
| Systemic lupus erythematosus | 6 | 20 | 4.77E-08 |
| Hashimoto thyroiditis | 5 | 11 | 5.81E-08 |
| Graves disease | 5 | 11 | 5.81E-08 |
| Eosinophilic granulomatosis with polyangiitis; Churg-Strauss syndrome | 5 | 11 | 5.81E-08 |
| Allograft rejection | 6 | 21 | 6.62E-08 |
| Sjogren syndrome | 5 | 12 | 9.88E-08 |
| Adult onset Still disease; Adult Still disease | 5 | 12 | 9.88E-08 |
| Systemic sclerosis; Systemic scleroderma | 6 | 23 | 1.21E-07 |
| Giant cell arteritis; Temporal arteritis | 5 | 13 | 1.59E-07 |
| Celiac disease | 5 | 13 | 1.59E-07 |
| Tuberculosis | 5 | 16 | 5.26E-07 |
| Asthma | 5 | 18 | 1.01E-06 |
| Combined oxidative phosphorylation deficiency | 7 | 61 | 3.90E-06 |
| Dilated cardiomyopathy | 6 | 52 | 1.88E-05 |
| Type 1 diabetes mellitus | 5 | 35 | 3.31E-05 |
| Methemoglobinemia | 3 | 7 | 4.04E-05 |
| Leigh syndrome | 3 | 18 | 0.000862 |
| Mitochondrial complex I deficiency | 4 | 42 | 0.001022 |
| Distal hereditary motor neuropathies | 3 | 21 | 0.001373 |
| Anemia due to disorders of nucleotide metabolism | 2 | 8 | 0.003019 |
| Retinitis pigmentosa | 5 | 92 | 0.003055 |
| Complex cortical dysplasia with other brain malformations | 3 | 30 | 0.003904 |
| Congenital disorders of glycosylation type I | 3 | 31 | 0.004289 |
| Spondyloepimetaphyseal dysplasia | 2 | 10 | 0.004784 |
| Anemia due to disorders of glutathione metabolism | 2 | 11 | 0.005807 |
| Cowden syndrome | 2 | 11 | 0.005807 |
| Microphthalmia, syndromic | 2 | 14 | 0.009407 |
| Charcot-Marie-Tooth disease; Hereditary motor and sensory neuropathy | 4 | 79 | 0.010092 |
| NK cell defects | 1 | 1 | 0.010615 |
| Adenylosuccinate lyase deficiency | 1 | 1 | 0.010615 |
| Orotic aciduria | 1 | 1 | 0.010615 |
| Pyogenic bacterial infections, recurrent, due to MYD88 deficiency | 1 | 1 | 0.010615 |
| Tumor necrosis factor receptor-associated periodic syndrome; Familial periodic fever | 1 | 1 | 0.010615 |
| Phosphoserine aminotransferase deficiency | 1 | 1 | 0.010615 |
| STAR syndrome | 1 | 1 | 0.010615 |
| Ethylmalonic encephalopathy | 1 | 1 | 0.010615 |
| Lathosterolosis | 1 | 1 | 0.010615 |
| Neutral lipid storage disease with myopathy | 1 | 1 | 0.010615 |
| Spondyloenchondrodysplasia with immune dysregulation (SPENCDI); Spondyloenchondrodysplasia (SPENCD) | 1 | 1 | 0.010615 |
| Costello syndrome | 1 | 1 | 0.010615 |
| Verheij syndrome; Chromosome 8q24.3 deletion syndrome | 1 | 1 | 0.010615 |
| Burn-McKeown syndrome | 1 | 1 | 0.010615 |
| Microhydranencephaly | 1 | 1 | 0.010615 |
| Hyperbiliverdinemia | 1 | 1 | 0.010615 |
| Pyruvate dehydrogenase E1-beta deficiency | 1 | 1 | 0.010615 |
